# Are Turkeys (*Meleagris gallopavo*) Motivated to Avoid Excreta-Soiled Substrate?

**DOI:** 10.3390/ani10112015

**Published:** 2020-11-02

**Authors:** Valerie Monckton, Nienke van Staaveren, Christine F. Baes, Agnese Balzani, Isabelle Y. Kwon, Peter McBride, Alexandra Harlander-Matauschek

**Affiliations:** 1 Department of Animal Biosciences, University of Guelph, 50 Stone Road E., Guelph, ON N1G 2W1, Canada; vmonckto@uoguelph.ca (V.M.); nvanstaa@uoguelph.ca (N.v.S.); cbaes@uoguelph.ca (C.F.B.); agnese.balzani@ucd.ie (A.B.); kwon@uoguelph.ca (I.Y.K.); pmcbri01@uoguelph.ca (P.M.); 2 Institute of Genetics, Vetsuisse Faculty, University of Bern, 3001 Bern, Switzerland

**Keywords:** welfare, *Meleagris gallopavo*, bedding, preference, operant methods, rubber mats, litter, floor, ground

## Abstract

**Simple Summary:**

Commercial turkeys are raised in large barns at stocking densities that cause excreta (or feces) to quickly accrue in the turkeys’ environment. Even though commercial turkeys spend most, if not all, of their time in contact with their excreta, we do not know how turkeys perceive this soiled environment. Therefore, our study used six pens of four turkeys, dividing each pen with a barrier that contained two one-way push-doors. This created two compartments: a “home” compartment containing soiled wood shavings, and a “treatment” (T) compartment containing fresh pine and spruce wood shavings (FP), soiled pine and spruce wood shavings (SP), ammonia reductant-treated soiled pine and spruce wood shavings (TSP), no substrate (NS), or a feed treatment. To establish the turkeys’ motivation to access these resources, we weighed the door to T with 0%, 20% or 40% of the turkeys’ body weight. The number of turkeys that pushed the maximum door weight was used as an indicator for their motivation. Additionally, time spent in T and the odds of visiting T were examined to determine how the turkeys responded to increasing challenge. We found that the turkeys preferred feed over all other resources and showed equal motivation for all floor substrate treatments.

**Abstract:**

The soiling of bedding on modern turkey farms combined with turkeys’ reduced ability and opportunity to perch and roost at elevation, forces them to spend most, if not all, of their time in contact with their excreta. To determine turkeys’ perspective on these conditions and the value they place on unsoiled bedding vs. soiled litter (collectively, substrates), we used twenty-four eleven-week-old turkey hens divided into six two-compartment pens. In the “home” compartment (H), we placed soiled wood shavings, while the “treatment” compartment (T) contained no substrate (NS), fresh pine and spruce wood shavings (FP), soiled pine and spruce wood shavings (SP), ammonia reductant-treated soiled pine and spruce wood shavings (TSP), or a feed treatment. One-way push-doors separated the two compartments. The door leading to T weighed an additional 0%, 20% or 40% of the turkeys’ body weight while the door to H remained unweighted. All birds were exposed to each resource and door weight combination in a systematic order. We measured the turkeys’ motivation based on the number of birds that pushed the maximum weight to access each resource, the amount of time spent in T, and the number of visits to T. Our findings show that turkeys worked harder to access feed compared to all the floor substrate treatments. Additionally, they were equally motivated to access all the substrate treatments.

## 1. Introduction

Unlike wild turkeys that spend their days foraging on the ground and roost in trees at night [1], modern-day domestic turkeys spend much of their lives on the ground. While turkeys have been shown to use perches at younger ages, their use decreases as turkeys get older and heavier, with none of the birds perching by 10 weeks of age [2]. Thus, providing perches is not a common practice among turkey farmers in Canada [3]. The lack of elevated perching and roosting areas combined with declining use with age and weight means that turkeys spend most, if not all, of their time in contact with the ground. Moreover, these animals are housed in barns at stocking densities that allow the floor (and any bedding on it) to quickly accrue with feathers, waste feed, and excreta. Standard litter management recommendations often prohibit the use of toxic beddings and require that litter be maintained at an adequate moisture level [4], but they do not require or recommend short-term cleaning schedules (e.g., weekly cleaning) to manage the degree of soiling in the barn [4,5]. As a result, combined brooder and grow-out barns often require that litter be cleaned out after every flock, while grow-out-only barns may require only yearly cleanings [5]. A recent survey of housing and management of turkey flocks in Canada, for which 20% of surveyed turkey farms responded, revealed that 15.7% of farmers kept multiple flocks on the same litter in grow-out barns [3]. Similarly, a 2007 study in Australia found that 27% of turkey farms did not clean out soiled litter between flocks, but 82% provided an additional fresh layer of bedding for these new flocks. While the unnaturalness of this environment may raise questions regarding the well-being of turkeys kept on litter, current practices can also affect their health.

Since turkeys have a longer grow-out period (~14 weeks) compared to other Galliformes raised for meat, like broiler chickens (~35–42 days), improperly managed litter can lead to more frequent and, occasionally, severe, health outcomes for the animals. In particular, as excreta builds up, substrate moisture content increases. High moisture content in bedding or litter (collectively, substrates) alone may cause contact dermatitis, either on the footpad or the breast area, which can lead to lesions and ulcers [6,7,8,9,10]. Contact dermatitis is painful [6] and is recognized as an important welfare issue by farmers [11]. This condition can be quite common, as 73–95% of farmed turkeys may show signs of footpad dermatitis [12,13] while up to 27% of male turkeys and 7% of female turkeys surveyed on German turkey farms exhibited breast lesions [9]. Apart from this, high moisture content also raises ammonia production [14], which increases the risk of keratoconjunctivitis and respiratory disease [15]. The adverse health outcomes associated with high ammonia led to the development of chemical ammonia reductants that trap ammonia in the litter as ammonium. Ammonia reductants can be used to reduce the incidence of ammonia-related disease in birds. Galliformes avoid environments with atmospheric ammonia concentrations exceeding 10 ppm [16], therefore, ammonia reductants could also help us determine if soiled environments by themselves—without ammonia—are aversive. However, these chemical ammonia reductants also act as acidifiers [17], and it is unknown whether or not turkeys would prefer to avoid them.

Given that soiling alone may impede turkeys’ ability to perform rewarding behaviors, such as dustbathing and foraging [18,19,20], it is possible they may prefer to avoid soiled environments. The higher moisture content of litter combined with compaction over time may cause litter to cake, reducing friability and, thus, the ability of the substrate to provide a satisfying dust bath. This, as well as resting on soiled bedding, may lead to soiled integument. Such soiling is unusual in wild birds, particularly in those that are healthy [21]. If turkeys find soiled environments aversive, then the frustration of being unable to escape a soiled environment may be compounded by their soiled integument.

Many countries require or recommend that turkeys be reared with substrate (e.g., Canada [4]) as it provides opportunities for foraging and exploring behavior [22]. However, Farghly et al. [23] showed that, in hotter climates, slotted (or slatted) floors reduced the incidence of disease in turkeys and helped to reduce body temperature, airborne dust particulates, and ammonia concentrations [23]. As such, whether or not turkeys show a relative preference for substrate or no substrate under the given experimental conditions could have enormous implications for their welfare.

In a laboratory setting, preference tests allow animals to show their relative preferences for different resources by spending more time with, more quickly approaching, or more frequently using one or more of the resources. Yet, preference tests alone cannot determine the value of different resources compared to one another. To ascribe preference and value, consumer demand motivation tests present with increasingly challenging obstacles to animals that they must overcome to access a resource [24,25]. Examples of these challenges include pecking a key [26,27], overcoming a barrier [28], or pushing a weighted door [29,30]. This study is the first to use weighted push-doors to assess turkeys’ motivation to access different substrate treatments, and used the percentage of birds that pushed the maximum door weight as a proxy for maximum price paid. This method measures motivation [31,32] by measuring the value of a resource to an animal through the maximum amount of work the animal will expend to access a resource [33]. Additionally, the turkeys’ ability to leave the treatment area without paying a cost meant that the reward size (length of visits) was under the turkeys’ control. Therefore, we also recorded the time spent in and the number of visits to each treatment with varying price. These metrics of motivation to access substrate treatments were then compared to the turkeys’ motivation to access feed, the gold standard of comparison [26].

Because of the unnaturalness of a soiled environment, as well as the ammonia it produces, we predicted that the turkeys would show greater motivation to access fresh pine and spruce shavings over soiled pine and spruce shavings (a commonly used bedding for turkeys in Canada [3]). We also predicted that the birds would prefer ammonia reductant-treated soiled pine and spruce shavings over soiled pine and spruce shavings, and that they would prefer substrate over no substrate. Thus, we predicted that the turkeys would differentiate between the substrate treatments in the following rank order, from most to least preferred: fresh pine and spruce wood shavings, ammonia reductant-treated soiled wood shavings, soiled wood shavings, and no substrate.

## 2. Materials and Methods

### 2.1. Ethical Approval

The University of Guelph Animal Care Committee (Animal Use Protocol Number 3169) approved this study before testing. Additionally, we followed the ARRIVE guidelines in the planning and conducting of this experiment [34].

### 2.2. Housing, Feeding, and Management

**Figure 1:**
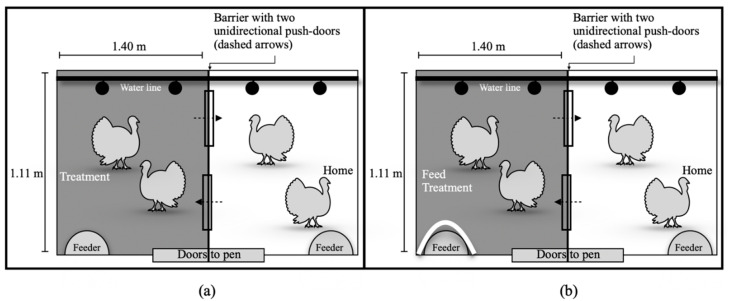
Pen layout with pen divider creating two different compartments (home and treatment) accessible through unidirectional doors. The treatment and home compartments alternated locations to avoid side bias. Each compartment had ad libitum feed and water access. (**a**) Pen setup to test substrate treatments (soiled pine and spruce wood shavings (SP), soiled pine and spruce shavings treated with an ammonia reductant (TSP), fresh pine and spruce wood shavings (FP), no substrate (NS)). (**b**) Pen setup for the feed treatment, which involved blocking access to feed in the home compartment.

We divided 24 9-week-old non-beak trimmed Hybrid XL turkey hens into groups of four across six different pens. Birds within a pen were differentiated by Twit-Stik^®^ livestock color sticks (blue, green, pink or red), in addition to their wing tags. The bottom of all six 1.11 m × 2.80 m floor pens (Figure 1) was laid with rubber mats covered in Pestell™ pine and spruce wood shavings. Feed and water were provided ad libitum, and the turkeys experienced light at 20 lux from 6:00 to 22:00 every day with 20 min of sunrise/sunset.

### 2.3. Experimental Design and Protocol

The motivation of turkeys to assess different substrates was assessed using a consumer-demand design where birds had to move through a weighted push-door to access substrate treatments. Each of the six pens were divided into a home (H) and treatment (T) compartment by a barrier that contained two unidirectional push-doors. Both compartments were identical and contained feeder and water lines (Figure 1). Turkeys were habituated to the experimental push-door set-up and substrates for a two-week period.

The experimental testing began when birds were 11 weeks old. During this time, the home compartment (H) always contained soiled litter that had been present within the pen during the habituation period. The treatment compartment (T) contained one of the four following substrate treatments: (1) fresh pine and spruce wood shavings (FP), (2) soiled pine and spruce wood shavings (SP) collected from the turkeys’ pens prior to the experiment, (3) soiled pine and spruce wood shavings treated with an ammonia reductant (37 kg/100 m^2^ of PLT^®^—Poultry Litter Treatment, Jones-Hamilton Co., United States of America) (TSP), or (4) no substrate (rubber mats over concrete floor; NS). Additionally, a fifth treatment was introduced as a gold standard [26], (5) feed, during which access to the feeder was only available in the T compartment and soiled litter was provided in both compartments. This was the only time that feed was not accessible in H, under all other treatments feed was ad libitum available in both H and T compartments. On the last day of trials, we sampled all substrate treatments at the start and end of the day for content analysis (SGS labs, Guelph, ON, Canada). The average moisture content (%), pH, and ammonium (%) concentration are reported in Table 1.

**Figure 2:**
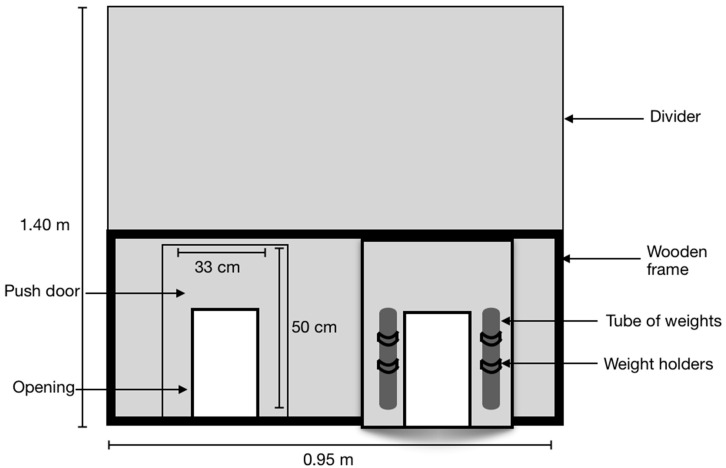
Divider layout with push doors viewed from turkey eye level. Dividers were built high (1.4 m) to ensure the birds would not perch or attempt to get over them. We equipped each divider with two push doors that mounted just below the wooden frame. Furthermore, we fixed each unidirectional push door with metal “weight holders” that held tubes of modifiable weight (modified by adding or removing lead weights). The doors were made of transparent plastic.

To measure the birds’ motivation to access a resource (feed, FP, SP, TSP, or NS), an equivalent weight of 0%, 20% or 40% of the turkey hens’ average body weight was mounted to the unidirectional door that led from H to T. Average body weight was determined weekly by weighing all birds in the pen and the 20% and 40% door weights were adjusted accordingly. Birds could freely return from T to H through another unidirectional door that was always mounted with 0% of their body weight (Figure 1 and Figure 2).

This led to a total of 15 door weight and resource combinations that were tested over the course of 4 weeks. Door weights and resources were changed daily according to a systematically varied schedule that allowed each pen of birds to experience each combination once. As such, a certain pen could experience the door weight and resource combination of, e.g., 40% door weight for feed in week 1, while another pen would experience this 40% door weight for feed in week 4 etc. until each pen had received all door weight and resource combinations once. When removing the previous resources, SP and TSP litters were stored, while FP was discarded, and the rubber mats from NS were cleaned. Birds started each trial day from the H compartment, and T and H compartments switched sides every day to prevent side bias. A camera (Samsung SNO-5084R, Samsung Techwin Co., Gyeonggido, Korea) was installed above each pen to record daily from 12:30–22:00 and 06:00–09:30. The amount of time turkeys spent in T and the number of times they visited T under each combination were determined via instantaneous scan sampling of the individual bird’s position in each pen at 30-min intervals for ~14 h (or 28 time points) per day.

Birds were inspected weekly to monitor for footpad dermatitis using the scoring system provided by the 2009 Welfare Quality^®^ Consortium [35]. This scoring method visually assesses footpad dermatitis severity on a 0–4 scale, where 0 is no evidence of footpad dermatitis, birds with score 1 and 2 are classified as minimal evidence of footpad dermatitis (minor lesions), and score 3 and 4 are considered as evidence of footpad dermatitis (large lesions) [35].

### 2.4. Statistical Analysis

Data were analyzed in SAS Studio (SAS Inst. Inc., Cary, NC, USA). To determine the turkeys’ motivation to access the five resources (feed, FP, SP, TSP, or NS), we examined the proportion of birds that pushed the maximum offered door weight (40%) for each resource in T. We then analyzed these data using a non-parametric chi-squared goodness-of-fit test that determined whether the observed proportions differed from the expected proportions for the five resources.

Generalized linear mixed models (GLIMMIX) were used to determine the effect of resource (feed, FP, SP, TSP, NS), door weight (0%, 20%, 40%), and their interaction on the odds of entering T and the percentage of time birds spent in T. Pen was considered as the experimental unit. As only a small proportion of the birds entered the T more than once, we created a categorical variable for entering T (bird entered T: 0 times, 1 time, or >1 time). A multinomial distribution was used to analyze the number of times a bird entered T, and odds ratios and 95% confidence interval (CI) are presented. The time spent in T was calculated for each hen based on the number of time points an individual was present in T out of a total observed time points. An arcsine transformation was used to meet the assumptions of normally distributed residuals and homogeneity of variance, which were examined graphically with QQ plots. The results are presented as backtransformed least square (LS) means ± SEM. Statistical significance was considered at *p <* 0.05, and tendencies are reported at 0.05 ≤ *p* ≤ 0.1.

## 3. Results

All turkeys began the experiment without footpad dermatitis. However, by the last week, 54.17% of them showed signs of footpad dermatitis (score > 0) on at least one of their feet.

### 3.1. Proportion of Birds that Pushed the Maximum Offered Door Weight (40%) to Access Each Resource

All birds pushed the maximum offered door weight (40%) for feed; however, not all birds pushed 40% to access the substrate treatments. Moreover, birds sometimes chose not to enter a substrate treatment at all (Table 2).

**Figure 3:**
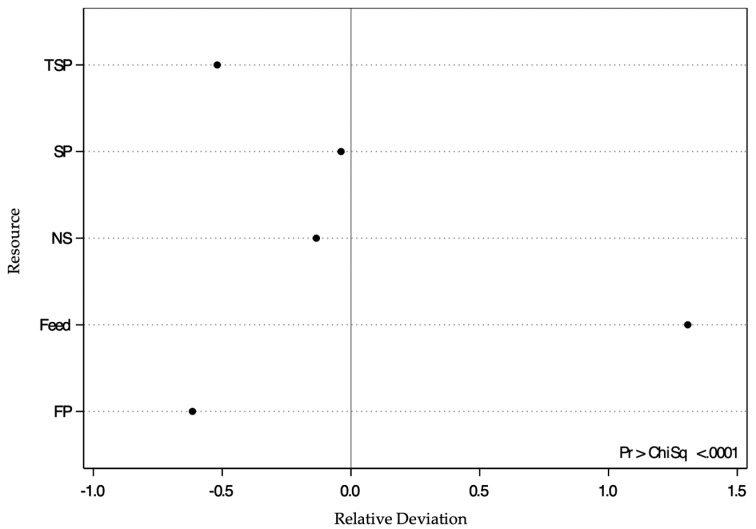
The relative deviation from the expected proportion of turkeys (*n* = 24; expected proportion indicated by the vertical line at 0.0) that pushed the maximum door weight (40%) to access each resource (feed, soiled pine and spruce wood shavings (SP), fresh pine and spruce wood shavings (FP), ammonia reductant-treated soiled pine and spruce wood shavings (TSP) and no substrate (NS)). If all the dots were on the line at 0.0, this would indicate that an equal proportion of birds pushed a maximum door weight of 40% to access all resources, implying that they ascribed equal value to all resources. A positive deviation indicates that a higher proportion of turkeys than expected pushed 40%, while a negative deviation indicates that a lower proportion of turkeys pushed the 40% than expected.

A chi-square test assessing the number of birds that pushed the maximum offered door weight (40%) to access each resource found that observed proportions differed from the expected proportions if all resources had equal value (Χ^2^ = 24.73, df = 4, *p <* 0.001). Specifically, it found that a larger proportion of turkeys than expected pushed the maximum offered door weight (40%) to access feed. This result is illustrated in Figure 3 as a positive deviation from the expected value (0.0 line), whereas a negative deviation indicates that a smaller proportion of birds than expected pushed 40% to access FP, TSP, NS and SP. However, the number of turkeys that pushed the maximum offered door weight was close to expected for NS and SP.

### 3.2. The Effect of Increasing Cost on Visits to and Time Spent in the Treatment Compartment

#### 3.2.1. Odds of Visiting the Treatment Compartment

Throughout the experiment, the turkeys chose not to enter the treatment compartment 60.83% of the time. When they did, they usually entered only once (27.50%). In total, the turkeys visited the T compartment 88 times to access the feed treatment, while they only visited each substrate approximately 29 times (data not shown). An overview of the number of turkeys that did not visit a resource, visited once or visited more than once is shown in Table 3.

**Figure 4:**
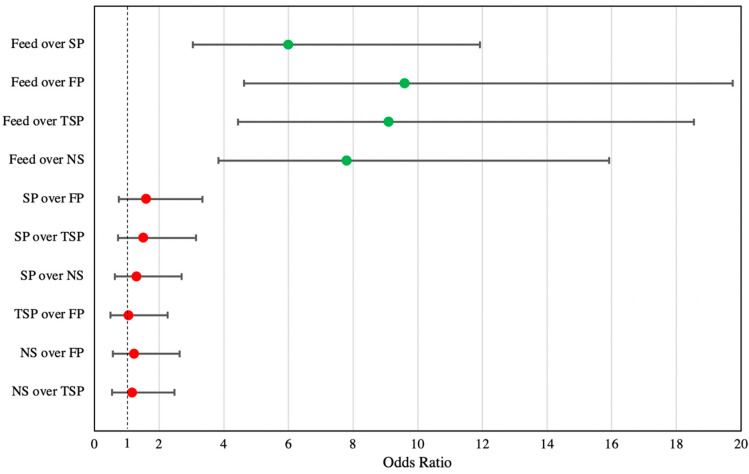
The odds of the turkeys entering the treatment compartment in the presence of one resource (feed, soiled pine and spruce wood shavings (SP), fresh pine and spruce wood shavings (FP), ammonia reductant-treated soiled pine and spruce wood shavings (TSP) and no substrate (NS)) over another. Odds ratios are represented by a solid dot and flanked by a 95% CI. Significant odds ratio comparisons (green dots) have a CI that does not overlap 1, while non-significant comparisons (red dots) have a CI that overlaps 1.

Whether or not turkeys chose to enter the treatment compartment was not affected by the interaction between door weight and resource (F_8,321_ = 1.02, *p* = 0.4236) or the door weight alone (F_2,321_ = 0.24, *p =* 0.7857). However, the resource present in T had a significant effect on the odds of entering T (F_4,321_ = 15.00, *p <* 0.0001). Specifically, the turkeys were more likely to enter T in the presence of feed compared to the substrate treatments, while they were equally likely to enter the different substrate treatments (Figure 4).

#### 3.2.2. Time Spent in the Treatment Compartment

**Figure 5:**
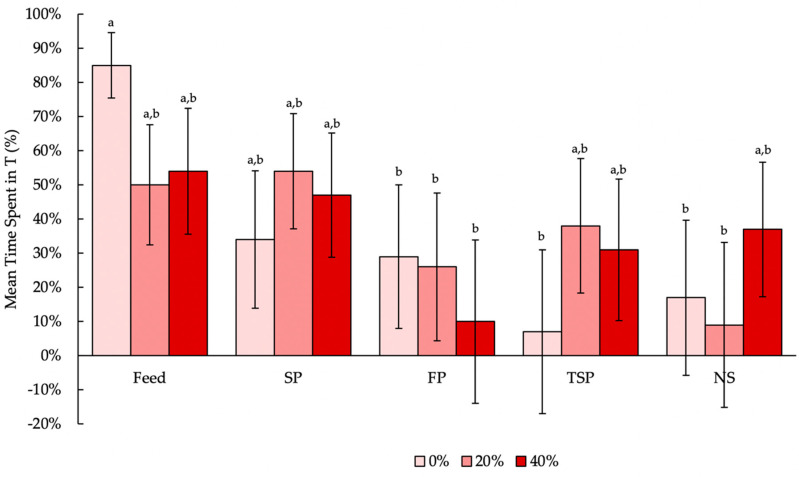
The interaction of door weight (0%, 20% or 40% of turkeys’ body weight) and resource (feed, soiled pine and spruce wood shavings (SP), fresh pine and spruce wood shavings (FP), ammonia reductant-treated soiled pine and spruce wood shavings (TSP) or no substrate (NS)) on the mean time spent (%; backtransformed LS means ± SEM) in the treatment compartment (T) for 14 h per day. Bars with different letters display significant differences (*p<* 0.05).

There was a significant interaction between resource and door weight (F_8,321.3_ = 2.45, *p* = 0.0137, Figure 5) largely because of the difference between time spent in the feed treatment compared to FP, TSP and NS. Hens spent significantly more time in T with the feed treatment at 0% door weight compared to TSP at 0% (t_321.1_ = 5.22, *p* < 0.0001), NS at 0% (t_321.1_ = −4.33, *p* = 0.0018) or NS at 20% (t_321.2_ = 4.95, *p* = 0.0001). Additionally, the hens spent more time with the feed treatment at 0% compared to FP at 0% (t_321.2_ = −3.48, *p* = 0.0414), 20% (t_321.3_ = 3.63, *p* = 0.0254) and 40% (t_321.3_ = 4.87, *p* = 0.0002).

**Figure 6:**
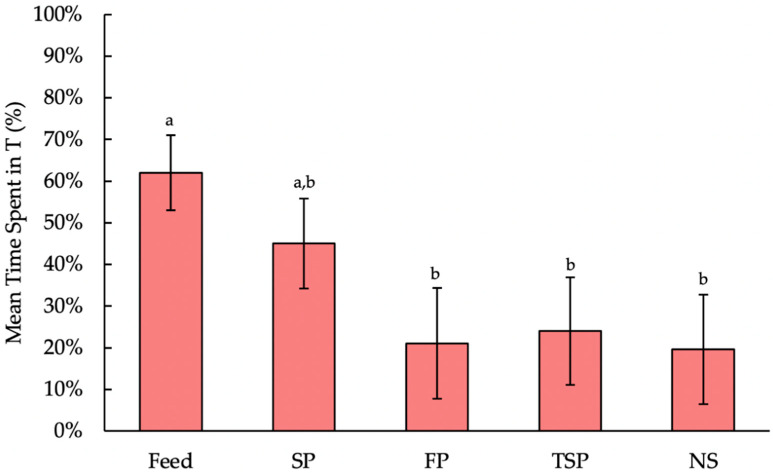
Effect of resource (feed, soiled pine and spruce wood shavings (SP), fresh pine and spruce wood shavings (FP), ammonia reductant-treated soiled pine and spruce wood shavings (TSP), no substrate (NS)) on the mean time spent (%; backtransformed LS means ± SEM) in the treatment compartment (T) for 14 h per day. Bars with different letters display significant differences (*p <* 0.05).

Moreover, because door weight did not affect time spent in T (F_2,321.2_ = 0.03, *p* = 0.9694), the interaction between door weight and resource is heavily influenced by resource, which significantly affected time spent in T (F_4,321.6_ = 7.74, *p* < 0.0001). Turkeys spent over 60% of their time in the feed treatment, which was more than the 20–25% of time they spent on FP (t_322.9_ = −4.26, *p* = 0.0003), TSP (t_322.4_ = 4.02, *p* = 0.0007) or NS (t_321.2_ = −4.65, *p* < 0.0001). However, they spent a similar amount of time on SP (t_321.5_ = −1.91, *p* = 0.3145) (Figure 6). There was no difference in the amount of time turkeys spent on any of the substrate treatments.

## 4. Discussion

This study used a two-compartment choice test to determine turkeys’ motivational strength for four different substrate treatments (SP, FP, TSP, NS) based on their responses to increased access costs (push-doors weighing an additional 0%, 20%, or 40% of the turkeys’ body weight). The turkeys’ motivation to access the substrate treatments was then compared to their motivation to access feed, the gold standard of comparison in motivation tests [26]. Motivation was assessed based on the proportion of birds that pushed the maximum offered weight (40%) to access each resource. This was used as a proxy for maximum price paid: a method that infers the value of a resource based on how much work an animal will expend to gain access to it [33]. We predicted that the turkeys would differentiate between the substrates in a rank order from most to least preferred: FP, TSP, SP, NS. We also hypothesized that increasing the cost to access the resources would reduce the number of turkeys’ visits to the treatment compartment (T) but would increase the time spent in T [33]. Our findings indicate that all the turkeys pushed the maximum offered weight (40%) for feed and that the proportion of birds that pushed 40% to access the substrate treatments was less than expected. The turkeys visited and spent more time in the treatment compartment when feed was present compared to the substrate treatments, except SP, which they spent a similar amount of time on compared to feed. Furthermore, they visited and spent the same amount of time in all the substrate treatments.

Feed was the only resource for which all turkeys pushed the maximum offered weight (40%). This result follows our predictions, and reinforces the high value of feed due to its physiological necessity [32]. Moreover, a smaller proportion of turkeys than expected pushed 40% to access FP, SP, TSP and NS, although this proportion was near to the expected value to access SP and NS. Hence, these findings agree with studies performed with laying hens [25,36] and broiler chickens [37] that found that Galliformes worked harder for feed than floor substrates. Additionally, selection for more muscle and faster growth rate [7] may mean that, similar to broilers [38], turkeys could have disrupted satiety mechanisms. In other words, turkeys may have a genetically increased motivation to eat [39], increasing their desire to access the feed treatment and to prioritize eating over other behaviors.

Additionally similar to broilers, it is possible that selection for greater size and growth rate has limited turkeys’ physical abilities [7]. Selection for larger breast muscle size may shift their center of gravity in a similar fashion to Corr et al.’s [40] findings with broilers. This shift in center of gravity could make rewarding behaviors such as foraging [41,42] and dustbathing [29,43] tiring or uncomfortable [7], which may be why a smaller proportion of birds pushed 40% to access substrates compared to feed. Furthermore, selection for more breast muscle can also lead to gait abnormalities, and place additional strain on the femur and tibiae, which may also deter turkeys from exploring and moving into the different substrate treatments [7,44]. Although gait was not scored in this experiment, over half the turkeys showed signs of footpad dermatitis by the end of the experiment. Previous work has shown that footpad dermatitis and gait score are correlated [12] and that under commercial conditions and higher stocking densities, up to 78–98% of female turkeys can be affected by footpad dermatitis by 12 weeks of age [45]. While the prevalence of footpad dermatitis was less in this experiment than on some farms [12,13], it could have reduced their motivation to move into T for anything aside from a necessity (feed).

While less turkeys pushed the maximum door weight than expected for most substrate treatments, they pushed for SP and NS close to the expected value if all substrates were equal. Therefore, it is possible that SP may be a more appealing substrate to perform rewarding behaviors—like dustbathing and foraging—when compared to the other options. Since resources were changed daily, SP was less caked compared to H litter, and was less acidic and smelled more familiar when compared to TSP [17]. Moreover, Moesta et al. [46] reported that, compared to fresh wood shavings (like FP), used wood shavings tended to have a smaller particle size and were found to be more stimulating and adequate for dustbathing in laying hens. Like dustbathing [29,43], chickens are also motivated to forage [41,42]. In particular, chickens prefer to forage in nutritive substrates [47] and, since cecal excreta contains vitamins, minerals and protein [48], they may find soiled substrates appealing to forage in. This could explain why chickens have been shown to forage in [49] and consume excreta [50,51]. Therefore, SP may have been a more satisfying substrate to both dustbathe and forage in compared to FP; however, this should be tested in a hypothesis-driven experiment.

Unlike other experiments that report birds preferring substrate over no substrate [24,52,53], this study found no difference in the way turkeys responded to substrate vs. no substrate. Unlike these other studies that examine chickens’ preferences for substrate compared to wire floors, this experiment used rubber mats for the no substrate (NS) treatment. To the authors’ knowledge, no experiments have examined turkeys’ preference for no substrate using non-wire floors. However, Farghly et al. [23] compared turkeys grown on litter to those grown on wire floors or rubber mats (among other floor types). While they did not perform preference tests, they found physiological differences in turkeys reared on wire floor compared to rubber mats, suggesting that rubber mats could be more comfortable. Therefore, given that NS was not significantly different from the other substrate treatments, other preference tests that compare Galliformes’ preferences for litter compared to wire floor [24,52,53] may not demonstrate the value of litter so much as the aversiveness of wire floor. Moreover, the Farghly et al. [23] found that birds reared on rubber mats had a nonsignificant but lower average body temperature compared to litter and wire floors. While our experiment found no significant effect of temperature on time spent, this could be due to the limited range of temperatures (22–28 °C). However, it should also be noted that the birds always had access to substrate in H. Therefore, we may have found that substrate had higher value if birds had to push to access substrate from an H compartment with no substrate.

In general, the turkeys preferred to stay in H regardless of door weight, and rarely moved into T more than once other than for the feed treatment. They were more likely to enter T in the presence of feed compared to any other treatment and subsequently spent more time in T with the feed treatment compared to FP, TSP and NS. Only with SP did turkeys spend the same amount of time as with feed. The turkeys’ low frequency of entering the substrates may have been affected by the presence of footpad dermatitis, which is a painful tissue injury [6]. Yet, they visited the feed compartment much more than any substrate treatment. Moreover, the turkeys spent about 60% of their time in the feed treatment, spending the other 40% of their time in H. Since the turkeys visited the feed treatment more often, and do not appear to stay very long, it appears that they did not avoid pushing the door. Therefore, rather than exhibiting an aversion to moving, the turkeys may have simply preferred the more familiar home compartment and visited the feed treatment when they were hungry. Alternatively, despite the habituation period, birds may have been fearful to enter and remain in a novel environment [54]. These factors may have contributed to the, relatively large standard errors were reported for the amount of time turkeys spent in the treatment compartment. It may also be explained by individual variation, with some turkeys being more exploratory or fearful, or simply seeking to obtain extra space or distance from conspecifics.

The finding that turkeys do not have a preference for any of the substrate treatments suggests that turkeys may be incapable of assessing the long-term consequences of contact with soiled litter [7,15,55,56]. This experiment yielded similar results to previous studies done with broilers [37] and laying hens [57]. However, preference tests must be meticulously designed to ensure that animals’ responses correspond to the experimenter’s question. The operand (pushing a door) used in this experiment has been validated and used with other Galliformes [29,30,58,59]. However, the push-doors may have been challenging for the turkeys to use due to their large breast muscles and the fact that our push-doors were originally designed for laying hens, which may have made pushing the doors awkward independent of door weight. Were this the case, it might explain why door weight did not significantly affect time spent or number of visits to T. This experiment may also have been limited by the environment, as the experiment room was not as well ventilated as in Monckton et al. [37]; therefore, the turkeys may have had more difficulty distinguishing between the less ammoniated substrate treatments (FP, TSP, NS). This may in turn have reduced the reward value of these treatments, complicating the task of pinpointing what exactly birds are motivated to access. Additionally, the turkeys were kept at a lower stocking density for a shorter period of time compared to a commercial environment, which reduced the build-up of excreta compared to a commercial environment. As such, while the moisture content of the fresh litter was similar to that of dry litter reported by others [60], the average moisture content was lower (approx. 30–40%) compared to the range of 50–70% reported by other studies [60,61]. The pH value of the fresh bedding likewise resembled that of dry litter as found in the literature, while both home and soiled litter were closer to pH values reported under commercial conditions [60]. Additionally, the home litter was used daily by the turkeys throughout the experiment. The soiled and ammonia reductant-treated litters were stored when not in use, while fresh bedding was discarded after 24 h of use. This could explain why the home litter and soiled litter had similar moisture, pH, nitrogen, and ammonia levels. Ammonia reductant-treated litter also showed similar moisture and nitrogen levels, though it also had a reduced pH and higher ammonium concentration, as expected. The relatively small differences between litter conditions could have influenced the turkeys’ ability to discriminate between the litters, and, therefore, further research under commercial conditions is required.

Additionally, this preference test used groups of animals, which makes observing individual preference more difficult, since Galliformes often make decisions as a group [62]. However, de Jong et al. [53] found that laying hens housed in isolation had greater difficulty learning a push-door task, so housing our turkeys in groups may have facilitated learning, in addition to being more natural. Moreover, since turkeys are not commonly reared in isolation, our choice to house them in groups could more accurately predict the choices they would make on-farm. As turkeys were housed in pens, only six replicates for each resource by door weight combination were available, which could be considered a limited sample size. However, retrospective power calculations revealed a power of 0.7 to determine differences between the time that turkeys spent in the fresh (FP) vs. soiled (SP) wood shavings. Yet, human perspective also limits this experiment, as we limited the choices that the turkeys could or could not be motivated to access. Moreover, our human perspective limits our interpretation of results, since humans instinctively view a soiled environment as undesirable, and cannot fully understand turkeys’ motivations. The individuality and personalities of each animal may also cause their motivation to vary as a result of different internal and external factors and as birds respond to challenges and a barren environment [63]. Whatever the true reason for the turkeys’ decisions in this experiment, we recommend that turkeys’ caretakers monitor and manage their environments with or without substrate as they seem to not avoid potentially unhealthy environments.

## 5. Conclusions

This is the first published study to assess the preference of turkeys for the presence of floor substrate and degree of its soiling. Turkeys preferred feed over all substrate treatments. More turkeys pushed the maximum door weight to access feed, followed by SP, NS, FP and TSP, suggesting that turkeys did not value all resources equally. They also spent more time in the treatment compartment when feed was present, although they spent the same amount of time when SP was present. However, the turkeys’ response to all of the substrate treatments was the same for time spent and odds of visiting the treatment compartment. Therefore, this study suggests turkeys may not exhibit a preference for management practices aimed at avoiding soiling of litter, reducing ammonia concentrations or providing fresh bedding. These findings may emphasize the responsibility of animal owners and/or farmers to diligently manage litter conditions for the birds’ health, as turkeys do not appear to avoid soiled or potentially harmful litter. However, further work is required to establish turkeys’ preferences for litter management practices under commercial conditions and to investigate the long-term effects of these practices on birds’ health and welfare.

## Figures and Tables

**Table 1:** Analysis of the average moisture content, pH, and ammonium concentration of the four litters and excreta in turkeys. Two samples were collected on the last experimental day, one collected immediately after placement and at the end of the day. No standard error is provided for excreta because only one sample was taken at the end of the day and because rubber mats were clean at placement.

Substrate	Average Moisture (%)	Average pH	Nitrogen (%)	Average Ammonium (ppm)
Home (H) litter	32.3 ± 7.34	7.9 ± 0.66	2.3 ± 0.27	8710.0 ± 3707.40
Fresh pine and spruce wood shavings (FP)	17.6 ± 13.24	6.6 ± 2.35	0.3 ± 0.09	1264.4 ± 626.69
Soiled pine and spruce wood shavings (SP)	39.6 ± 9.18	8.4 ± 0.44	1.8 ± 0.35	8290.3 ± 2697.75
Ammonia reductant-treated pine and spruce wood shavings (TSP)	31.4 ± 3.90	2.8 ± 1.34	2.3 ± 0.21	11,178.3 ± 4948.94
No substrate (NS) (excreta collected once)	40.3	5.4	5.1	4732.3

**Table 2:** The number of birds (*n*_total_ = 24) that were willing to push 0%, 20% or 40% as the maximum door weight to access each resource (feed, soiled pine and spruce wood shavings (SP), fresh pine and spruce wood shavings (FP), ammonia reductant-treated soiled pine and spruce wood shavings (TSP) and no substrate (NS)). Only the highest door weight that each bird pushed is presented.

	Maximum Door Weight Pushed							
	Did Not Enter		0%		20%		40%	
Resource	*n*	%	*n*	%	*n*	%	*n*	%
Feed	0	0.00	0	0.00	0	0.00	24	100.00
SP	9	37.50	0	0.00	5	20.83	10	41.67
FP	9	37.50	4	16.67	7	29.17	4	16.67
TSP	12	50.00	2	8.33	5	20.83	5	20.83
NS	8	33.33	0	0.00	7	29.17	9	37.50

**Table 3:** The number of turkeys that did not visit a resource, visited once or visited more than once for each resource (feed, soiled pine and spruce wood shavings (SP), fresh pine and spruce wood shavings (FP), ammonia reductant-treated soiled pine and spruce wood shavings (TSP) and no substrate (NS)) and door weight (0%, 20% or 40% of the birds’ bodyweight) combination.

Resource	Door Weight	No Visit		1 Visit		>1 Visit	
		*n*	%	*n*	%	*n*	%
Feed	0%	0	0.00	15	62.50	9	37.50
	20%	4	16.67	12	50.00	8	33.33
	40%	0	0.00	16	66.67	8	33.33
SP	0%	12	50.00	9	37.50	3	12.50
	20%	8	33.33	14	58.33	2	8.33
	40%	9	37.50	11	45.83	4	16.67
FP	0%	12	50.00	8	33.33	4	16.67
	20%	13	54.17	10	41.67	1	4.17
	40%	17	70.83	6	25.00	1	4.17
TSP	0%	17	70.83	3	12.50	4	16.67
	20%	11	45.83	12	50.00	1	4.17
	40%	13	54.17	7	29.17	4	16.67
NS	0%	15	62.50	8	33.33	1	4.17
	20%	14	58.33	4	16.67	6	25.00
	40%	10	41.67	14	58.33	0	0.00
